# Case of Injury of the Scrotum and Perineum

**Published:** 1855-05

**Authors:** Robert W. Gibbes

**Affiliations:** Columbia, South Carolina


Case of Injury to the Scrotum and Perineum. By Robert W. Gibbes, Jr., M. D., of Columbia, South Carolina.—Henry, colored man, aged 22 years, of robust constitution, and a workman at the foundry of J. N. Scoffield, while standing with one foot on a ladder and the other on a joist, engaged in adjusting a belt, on the 24th of July last, had his coat (which was buttoned closely in front) caught by the shafting, and his clothes and genital organs torn off.

I got to him about 8 A. M., which was within twenty minutes after the occurrence of the accident, and found that the following injury had been sustained:—The entire scrotum was torn off, with the skin and cellular tissue from two and a half inches above the spine of the pubis down to the edge of the sphincter and including all the breadth of the perineum, together with the left testicle with five inches of its cord attached, and all the integument and cellular covering of the penis, except a rim nearly half an inch wide at the extremity and continuous with the mucous membrane of the perpuce.

I picked up these parts (all in one piece) in a distant corner of the establishment where they were lying wrapped up in the cloths, and I have preserved the specimen in alcohol.

The patient was found lying on a table closely covered up with a blanket, (for his clothes with the exception of shirt, socks and neck handkerchief, had been all torn off), and suffering most acutely. Chloroform was immediately administered, and on examination the right testicle was seen hanging by its bare cord, and apparently covered only by the tunica vaginalis communis as high as the abdominal ring, where the elastic feel of the intestines was distinctly perceptible, as well as on the opposite side, where the end of the cord was so retracted as to render its ligation impossible.

I do not think more than a half ounce of blood could have been lost, as scarcely any oozing was observed, and the parts separated were almost as clean as if they had been sponged.

The gap in the perineum was brought together by three points of the interrupted suture, and, as it was impossible to form a covering for the raw surface and remaining testicle, a simple lint dressing was applied, the penis supported on the left thigh, and the testicle on a small bran cushion placed between the legs. The patient was then left, still under the influence of chloroform.

October 1st.—The patient now begins to walk about; (a little more than two months after the accident) and it is very interesting to observe nature's slow, steady process of reparation. The testicle is high up in the right inguinal canal, and being entirely covered, resembles somewhat in appearance a bubo. The perineum is completely closed, very nearly up to the penis, which is much diminished in length, and is gradually being covered by an apparently almost inextensible integument, similar, as might have been anticipated, to the cicatrix formed after a burn. The man, however, has occasional erections, and says that the organ swells out, stands nearly straight, and pains him but very little.

Not long after, this cicatrization being almost complete, the patient went out to his usual occupation.

January 28, 1855.—I have seen Henry to-day; the testicle, instead of resting as it did when I last saw him on the os pubis, can be felt very distinctly below and outside of the external ring. It is of about the natural size and appears by its weight to have pushed down the new skin and cellular tissue before it, thus forming for itself a thick and efficient scrotal envelope. The soft pubis has, as it were, descended or grown down upon the penis being continuous directly with the original mucous membrane of the prepuce, which last has, of course, lost its former characteristics and taken on those of skin; there being no prepuce now and the glans penis perfectly bare. The sexual appetite, he says, is as great as before the accident, and coition as agreeable as ever; the only difference he observes is that the semen "does not come as free." One of his fellow workmen said to me, "Oh he is as good a man as ever," to which Henry replied *that he believed he was.*

Though I have made every reference in my power, I have been able to find but two cases which bear any analogy to the above. In the Parisian Chirurgical Journal of Dessault for 1794, there is a case from Richter, translated into French from the German, by M. Brewer. A peasant's genital organs were torn off by his clothes being caught in a carriage wheel in the year 1782. The urethra and penis torn off as far as the prostate which "contused and torn, adhered by a few fibres and hung out of the wound." No vestige remained of either the right testicle or scrotum. Wound was dressed, patient bled and ordered a nitrated drink; pain and fever not very considerable; urine, which was suppressed for thirty-six hours, flowed involuntary and in great abundance; but the power of retaining it for six hours or even more soon recovered. On the fifteenth day the spermatic cord was divided near the abdominal ring; it as well as the testicle was found perfectly healthy. The prostate remained for some time so sensitive that the patient could not bear the slightest touch. At length, however, it got buried in the wound. After beginning to walk, any intemperance would occasion retention of urine which was relieved by purging. In ten weeks from the time of the accident, cicatrization was perfect. There remained an opening for the passage of the urine, three lines in diameter under symphisis pubis and a little to the left side.

The other case is related in Larrey's Memoirs of the Austrian Campaign of 1811. A soldier standing with his legs apart was struck from behind by a bullet which carried away the following parts, viz:—The margin of the sphincter ani, skin of the perineum, the bulbous portion of the urethra, some of the skin of the scrotum and the right testicle—dividing its spermatic cord close to the ring and tearing the skin of the penis and prepuce. He was left for dead on the battle field, and only some days after he was carried to Reneveck Hospital in a very feeble condition. A good deal of disorganized substance had to be excised, which caused some hemorrhage, necessitating the ligation of several branches of the pubic artery besides that of the spermatic. Baron Larrey covered the left testicle under the portion of dartos that remained and brought the edges together. After some grave and tedious complications, a cure was effected at the end of four months—one of the most remarkable, says M. Larrey, with which he is acquainted. Unfortunately the observation is very incomplete.—*Charleston Med. Jour.*

